# Asymmetric Supercapacitors Based on Hierarchically Nanoporous Carbon and ZnCo_2_O_4_ From a Single Biometallic Metal-Organic Frameworks (Zn/Co-MOF)

**DOI:** 10.3389/fchem.2020.00719

**Published:** 2020-09-23

**Authors:** Da He, Yu Gao, Yucen Yao, Ling Wu, Jiang Zhang, Zheng-Hong Huang, Ming-Xi Wang

**Affiliations:** ^1^Key Laboratory for Green Chemical Process of Ministry of Education, School of Chemical and Environmental Engineering, Wuhan Institute of Technology, Wuhan, China; ^2^College of Chemistry and Environmental Engineering, Chongqing University of Arts and Sciences, Chongqing, China; ^3^Hubei Province Key Laboratory of Coal Conversion and New Carbon Materials, School of Chemistry and Chemical Engineering, Wuhan University of Science and Technology, Wuhan, China; ^4^Laboratory of Advanced Materials, School of Materials Science and Engineering, Tsinghua University, Beijing, China

**Keywords:** metal-organic frameworks, asymmetric supercapacitors, One-for-All, nanoporous materials, ZnCo_2_O_4_

## Abstract

Metal-organic framework (MOF)-derived nanoporous carbons (NPCs) and porous metal oxide nanostructures or nanocomposites have gathered considerable interest due to their potential use in supercapacitor (SCs) applications, owing to their precise control over porous architectures, pore volumes, and surface area. Bimetallic MOFs could provide rich redox reactions deriving from improved charge transfer between different metal ions, so their supercapacitor performance could be further greatly enhanced. In this study, “One-for-All” strategy is adopted to synthesize both positive and negative electrodes for hybrid asymmetric SCs (ASCs) from a single bimetallic MOF. The bimetallic Zn/Co-MOF with cuboid-like structures were synthesized by a simple method. The MOF-derived nanoporous carbons (NPC) were then obtained by post-heat treatment of the as-synthesized Zn/Co-MOF and rinsing with HCl, and bimetallic oxides (ZnCo_2_O_4_) were achieved by sintering the Zn/Co-MOF in air. The as-prepared MOF-derived NPC and bimetallic oxides were utilized as negative and positive materials to assemble hybrid ASCs with 6 M KOH as an electrolyte. Owing to the matchable voltage window and specific capacitance between the negative (NPC) and positive (ZnCo_2_O_4_), the as-assembled ASCs delivered high specific capacitance of 94.4 F/g (cell), excellent energy density of 28.6 Wh/kg at a power density of 100 W/kg, and high cycling stability of 87.2% after 5,000 charge-discharge cycles. This strategy is promising in producing high-energy-density electrode materials in supercapacitors.

## Introduction

With the rapid development of electric vehicles and the popularity of portable mobile electronic devices, energy storage devices are urgently required to have the following merits simultaneously: high energy density, high power density, and a long cycle life. The two primary energy storage devices, batteries and supercapacitors (SCs), have their own pros and cons. Batteries deliver high energy density and efficiency but relatively low power density determined by their energy storage mechanisms (Zhou et al., 2016; Chen et al., 2017; Wang et al., 2017; Sun et al., 2020). Meanwhile, SCs exhibit high power density and a long cycle life with low energy density (Shaibani et al., 2017; Cai et al., 2018; Shao et al., 2018; Yi et al., 2020). Consequently, the traditional single energy storage devices (batteries or SCs) cannot meet all the application requirements.

Asymmetric Supercapacitors (ASCs) are promising hybrid devices that possess the merits of both batteries and SCs (Dubal et al., 2015; Sun et al., 2015; Guo et al., 2020), and can meet the demands for powering electric vehicles and other future multifunctional electrics. Thus, constructing such asymmetric supercapacitors has recently become the focus of SC research. In general, ASCs are composed of battery- and capacitive-like materials as positive and negative electrodes (Choudhary et al., 2017; Xu et al., 2017b), and their performance is highly dependent on the electrode materials. However, it is not easy to find the proper electrode materials for ASCs, because the required materials must not only deliver excellent electrochemical performance but also match well with each other (Zhao et al., 2018). Up to now, most reports on ASCs are concerned with the development of a positive electrode only; various metal oxides have been rationally designed, fabricated, and used as positive electrode materials (Choudhary et al., 2017; Ranjithkumar et al., 2020; Zhu et al., 2020). In most instances, activated carbon (AC) has been employed as the negative electrode, and the asymmetric performance of such ASCs was not ideal. Therefore, developing novel well-matched positive/negative electrode materials is key for obtaining high-performance ASCs.

Metal-organic frameworks (MOFs) (Zeng et al., 2019; Ji et al., 2020), also called porous coordination polymers, are a novel kind of crystalline porous material with periodic network structures generated by organic ligands and metal ions of clusters, and they have gathered considerable interest due to their tailored structure, large specific surface area (SSA), and multifunctional properties. Based on their inherent merits, potential applications of MOFs, and their derived materials have been investigated in many fields. For example, the porous CuO/Cu_2_O heterostructure derived from Cu-MOFs inherited polyhedral morphology has a high surface area and large pore volume, and exhibited high activity in selective catalytic reduction of NO_x_ by NH_3_ at low temperatures (Wang et al., 2019b). Besides being used as catalysts (Lee et al., 2009), MOFs can also act as potential adsorbents (Zhang et al., 2019), as a stationary phase in chromatographic separation (Zhang and Chen, 2017), or in electrochemical detection (Zhang et al., 2018) and photocatalytic degradation of organic pollutants (Li et al., 2020), and etc. Recently, the exploration of MOFs in energy storage and conversion has gained more and more importance (Wang et al., 2018, 2019a; Xie et al., 2018). However, pristine MOFs usually have low electric conductivity, weak structure flexibility, and ion insertion steric hindrance, so the electrochemical performance of pristine MOF-based energy storage devices is not ideal. Then, rather than their direct use, MOFs are generally used as precursors to derive different porous nanomaterials, such as nanoporous carbons (NPC) (Salunkhe et al., 2016) and metal oxides (Salunkhe et al., 2017), by various thermal and/or chemical treatments. For example, nitrogen-doped NPCs were synthesized by the thermal decomposition of zeolitic imidazolate frameworks (ZIF-8) (Xu et al., 2017a), and the functionalized NPCs exhibited an enhanced overall capacitance. Hollow NiCo_2_O_4_ nanowall arrays were obtained from a two-dimension cobalt-based MOF (Guan et al., 2017), showing remarkable electrochemical performance when used as a flexible supercapacitor electrode material.

More interestingly, using MOFs as precursors for electrode materials in supercapacitors has another unique advantage, that is, the negative electrode materials (NPC) and positive electrode materials (metal oxides) can be obtained from the same single MOFs via the “One-for-All” strategy (Qu et al., 2018). Because both the negative-/positive electrodes derive from the same nanoarchitectures, they can match well-naturally, which is especially attractive in ASCs for they often suffer from poor-matched negative/positive electrode materials (Wang et al., 2019a). Based on the “One-for-All” strategy, Yamauchi Y. et al. (Salunkhe et al., 2015b) fabricated NPCs and cobalt oxide (Co_3_O_4_) from a single MOF (ZIF-67); the assembled ASCs with the ZIF-67-derived NPCs and Co_3_O_4_ showed a high energy density of 36 Wh kg^−1^. Zou R. et al. (Qu et al., 2018) synthesized positive and negative electrode materials for ASCs from a single pillared MOF; the assembled ASCs displayed a wide voltage window of 1.5 V and high energy density. In comparison with the one-metallic MOFs, mixed-metallic or bimetallic MOFs presented with a better performance (Jiao et al., 2017), because bimetallic MOFs could provide rich redox reactions deriving from improved charge transfer between different metal ions, so their supercapacitor performance could be greatly enhanced.

Herein, inspired by the “One-for-All” strategy, we synthesized NPC and bimetallic oxides from a single bimetallic Zn/Co-MOFs. The as-synthesized MOF-derived NPC and bimetallic oxides were utilized as negative and positive materials to assemble hybrid asymmetric supercapacitors (ASCs) with 6 M KOH as electrolyte. The capacitive performance of NPC and bimetallic oxides were investigated both in a three-electrode system and in an ASC device.

## Experimental Section

### Synthesis of Zn/Co-MOF and Its Derived NPC and Bimetallic Oxides

All the chemicals were purchased from commercial companies and used without further purification. In a typical synthesis procedure, methylimidazole (MIM, 1.968 mg, 24 mmol) was dissolved in methanol (200 mL). Zn (NO_3_)_2_·6H_2_O (0.594 g, 10 mmol) and Co (NO_3_)_2_·6H_2_O (1.164 g, 20 mmol) were dissolved in methanol (200 mL) to form a solution. Then, they were mixed rapidly and magnetically stirred for 24 h at room temperature. Next, the purple suspension solution was centrifuged, and the sediment was washed with methanol five times. The resulting powders were dried at 80°C for 12 h to obtain Zn/Co-MOF.

NPC and ZnCo_2_O_4_ were both prepared from the as-prepared Zn/Co-MOF. To obtain MOF-derived NPC, the Zn/Co-MOF was heated to 900°C at a ramping rate of 5°C min^−1^ and kept for 2 h in an N_2_ atmosphere, then cooled to room temperature. The sintered samples were washed with hydrochloric acid (1 M) to remove Zn and Co elements, and dried at 80°C for 12 h to obtain NPC. For the synthesis of ZnCo_2_O_4_, the Zn/Co-MOF powders were firstly annealed in 450°C for 1 h at a heating rate of 5°C min^−1^ under an N_2_ atmosphere, then the nitrogen supply was switched off, and both ends of the furnace were kept open to air at this temperature for an additional 1 h, leading to the conversion of these powders to oxides. The sample was designated as ZnCo_2_O_4_. In contrast, the Zn/Co-MOF was heated at 450°C for 1 h under N_2_ without further heat-treatment in air; the as-obtained sample was denoted as C@ZnCo_2_O_4_ because it contained a large amount of carbon.

### Characterization

Field emission scanning electron microscopy (FE-SEM, GeminiSEM 300) was employed to observe the morphology of the MOFs and their derivates. Element analysis was achieved with Energy dispersive X-Ray spectroscopy (EDX) at the K-edge of Zn, Co, O, and C. The crystalline structures of all the samples were characterized by X-ray diffractometer (Bruker D8 Advance, Cu K, λ = 1.5406Å). Nitrogen adsorption-desorption isotherms were obtained in an ASAP physisorption apparatus at 77 K. According to the British Standard BS ISO 9277-2010 (International Organization for Standardization. Technical Committee ISO/TC 24. Particle characterization including sieving, S.S., Particle characterization, 2010) and the nitrogen adsorption isotherms data in the relative pressure P/P_0_ range, the specific surface area (SSA) was calculated by applying the multi-point BET (Brunauer-Emmett-Teller) method. The pore size distribution (PSD) and pore volume were determined from the N_2_ adsorption/desorption isotherms based on the non-local solid density functional theory (NLDFT) model (Olivier, 1995; Wang et al., 2015, 2016) with an assumption of slit pore type.

### Electrochemical Measurements

The electrochemical capacitive performance of the MOF-derived electrode materials and ASC devices were tested and evaluated in 6 M KOH aqueous electrolyte solution under ambient conditions on a CHI760e electrochemical workstation (Shanghai ChenHua Instruments Co., China) with a three-electrode system. The working electrode was prepared by homogeneously mixing the as-prepared NPC or metal oxides, acetylene black, and polytetrafluoroethylene (PTFE, 60% suspension) with a weight ratio of 90:5:5 in ethanol to form a slurry. The slurry was then coated onto the nickel foam (1 × 1 cm^2^) current collector and dried at 80°C for 12 h in a vacuum oven. Hg/HgO and a platinum foil electrode were employed as the reference and counter electrodes, respectively. The mass loading of the active materials was controlled at about 3.0 mg/cm^2^. Supercapacitors were assembled by sandwiching filter paper between two symmetrical working electrodes in a two-electrode cell configuration (CR2025-type coin cell).

In order to evaluate the electrochemical properties of the samples, cyclic voltammetry (CV), galvanostatic charge–discharge (GCD), and electrochemical impedance spectroscopy (EIS) techniques were performed in the voltage window of −1 to 0 V and a frequency range from 10 mHz to 100 kHz. The gravimetric specific capacitance (C_g_, F/g) of a single electrode in three-electrode system was measured in 6M KOH. The gravimetric specific capacitance (C_cell_, F/g) in a two-electrode system were calculated on the basis of GCD curves. The energy density, E_cell_ (Wh/kg), and power density, P_cell_ (W/kg) of the active materials in the two-electrode system supercapacitors were also derived from GCD curves. The calculation formula is listed as follows:

(1) $$\begin{array}{l} C_{\text{g}}=\left(I\Delta t\right)/\left(m\Delta V\right) \end{array}$$

(2) $$\begin{array}{l} C_{\text{cell}}=I\Delta t/\left(M\Delta V\right) \end{array}$$

(3) $$\begin{array}{l} E_{\text{cell}}=C_{\text{cell}}\Delta V^{2}/\left(2\times 3.6\right) \end{array}$$

(4) $$\begin{array}{l} P_{\text{cell}}=3,600\times E_{\text{cell}}/\Delta t \end{array}$$

where I (A) is the current applied, Δt (s) and ΔV (V) are the discharging time and potential range after the IR drop, m (g) is the total mass of active material on the electrode, and M (g) is the total mass loading of the active materials on the two electrodes.

## Results and Discussions

**Figure 1:**
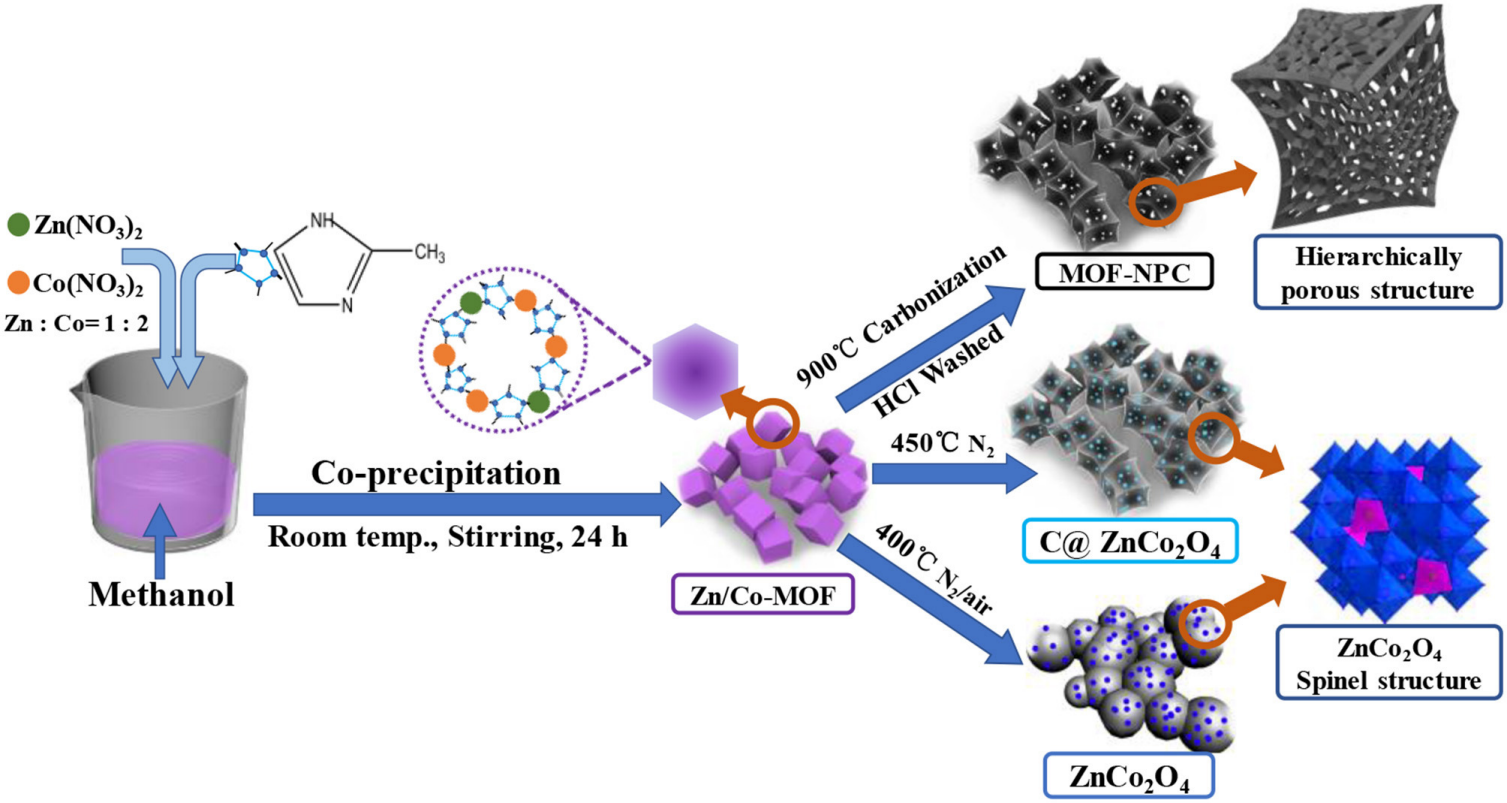
Schematic illustration for the synthesis of Zn/Co-MOF-derived nanoporous carbons (NPCs) and bimetallic oxides via the “*One-for-All*” strategy.

In this work, bimetallic MOF-derived electrode materials were synthesized via the “*One-for-All*” strategy, and the schematic illustration for the experimental processes is shown in Figure 1. The synthesis process involves two main steps. In the first step, the bimetallic Zn/Co-MOFs with a polyhedral shape was formed by the co-precipitation of Zn and Co ions in the presence of MIM in methanol at room temperature. The precipitated sample was denoted as Zn/Co-MOF. To obtain ZnCo_2_O_4_, the mole ratio of Zn to Co was controlled at 1:2. Second, after the first step, the as-synthesized Zn/Co-MOFs served as the precursor for producing both NPC and ZnCo_2_O_4_, which was achieved by directly pyrolyzing the Zn/Co-MOF under N_2_ atmosphere at 900 and 450°C, respectively.

**Figure 2:**
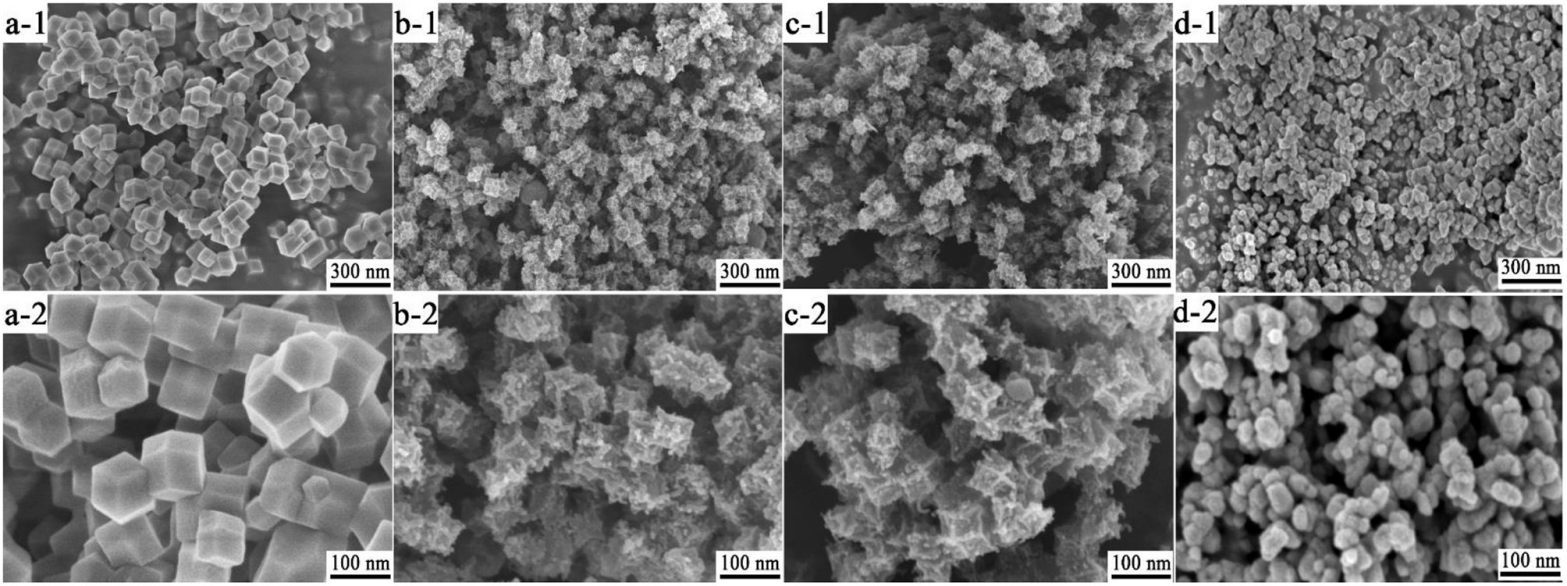
SEM images of low (-1) and high- magnification (-2): **(a)** Zn/Co-MOF; **(b)** MOF-NPC; **(c)** C@ ZnCo_2_O_4_; **(d)** ZnCo_2_O_4_.

The morphology, composition, and textural structures of the as-synthesized hierarchical porous MOFs and their derivates were characterized by various techniques. According to Figure 2, the pure Zn/Co-MOF nanocrystal particles (Figure 2A) had regular and high-symmetry geometry with good uniformity and displayed a relatively clean and smooth surface. Interestingly, the as-synthesized MOFs were of nanoscale size; the polyhedral edge length was about 80 nm, which is less than that reported in the literature (Niu et al., 2017). As for NPCs, derived from the pyrolysis of Zn/Co-MOF at 900°C followed by rinsing with HCl, they displayed similar morphologies to Zn/Co-MOF (Figure 2B), while there were some distortions in their geometry with a rough surface. Moreover, some obvious pores can be observed. The products obtained by the pyrolysis of Zn/Co-MOF at 450°C for 1 h just under nitrogen shown in Figure 2C had similar morphologies to the MOF-NPC, although some slight distortions can be observed in their geometry. They also retained the pristine polyhedral shape with regular edge and surface irregularity. By contrast, the products by pyrolysis of Zn/Co-MOF at 450°C under N_2_ followed in air depicted in Figure 2D shows obvious structural deformation; the polyhedral shape turned into a sphere-like shape, which was caused by the heat-treatment in air leading to some collapses in their structures. The original Zn/Co-MOF may be converted to metal oxides at 450 ^o^C in air. Their element compositions and crystal structures will be further evaluated.

**Figure 3:**
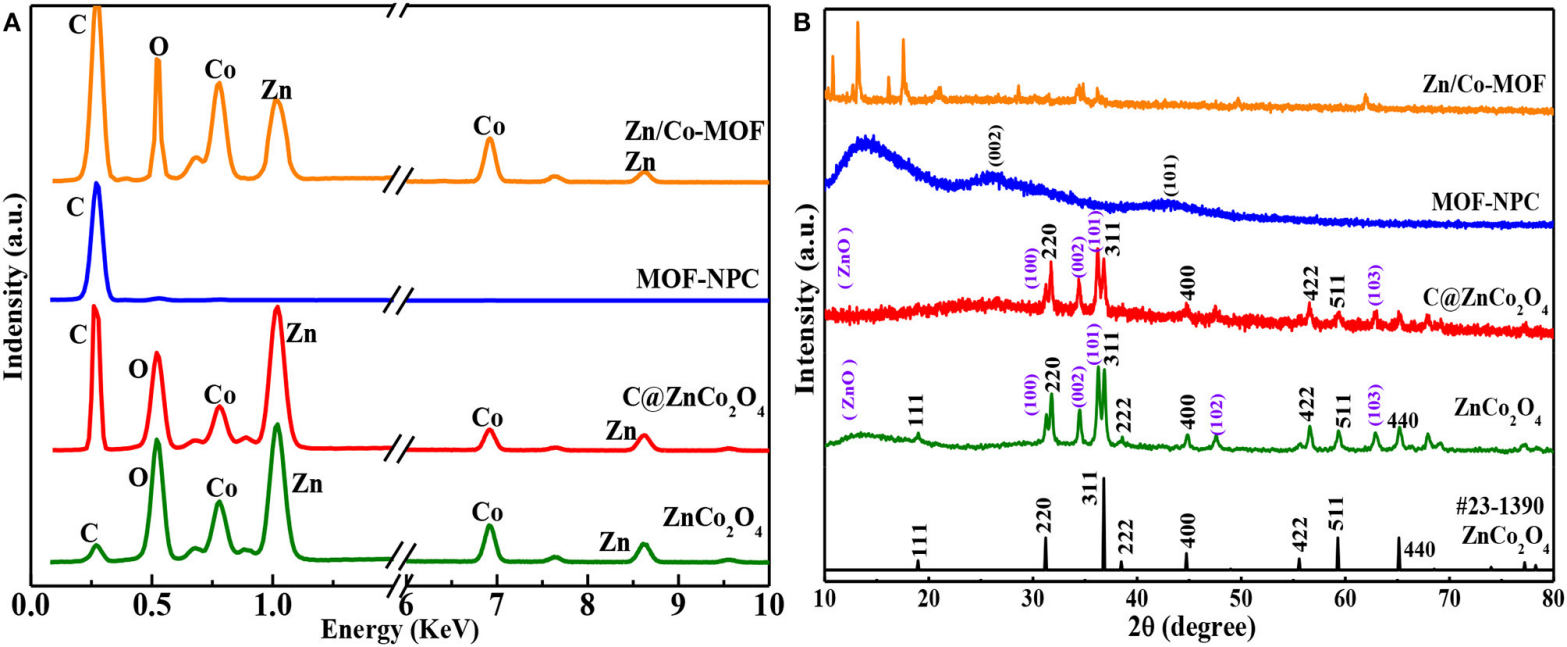
The EDX spectra **(A)** and XRD patterns **(B)** of the Zn/Co-MOF, MOF-NPC, C@ZnCo_2_O_4_, and ZnCo_2_O_4_.

The chemical compositions of Zn/Co-MOF, NPC, and ZnCo_2_O_4_ were confirmed by energy-dispersive X-ray spectroscopy (EDS), which are presented in Figure 3A. The EDS shows that C, O, Co, and Zn exist in the sample of Zn/Co-MOF, indicating the bimetallic MOFs were successfully prepared and that both Zn and Co elements were incorporated in the polyhedron. In the EDS of MOF-NPC, a strong peak of C is clearly observed with a very weak peak of O, while the peaks of Zn and Co cannot be found. This implied that nanoporous carbon materials had been formed containing negligible metal contents, by way of the high-temperature sintering and rinsing with HCl and water. When the MOF was annealed at 450°C only under nitrogen, all the elements are present in abundance; the relative content of metal elements relatively increased and that of C element decreased compared with the pristine MOF. As the annealed MOF was further heat treated at 450°C in air, the peak of C was very weak while the peaks of Zn and Co were strong, which suggested that the content of C reduced drastically and the metallic elements were converted to metal oxides. At 450°C in nitrogen, the carbonaceous components in MOFs cannot be carbonized into conductive carbon. But in the presence of oxygen in air, most of them will be burned off and more pure metal oxides will be produced. Therefore, the heat-treatment in air is essential for the enhancement of the final ASCs' electrochemical performance that were assembled by the MOFs-derived NPC and metal oxides.

The microcrystalline structures and phase purity of the as-prepared MOF and MOF-derived materials were confirmed by X-ray diffraction (XRD) and the results are shown in Figure 3B. The Zn/Co-MOF show similar XRD patterns to that of simulated ZIF-8 and Zn-Co-ZIF-0.5, including both the peaks' positions and relative intensities (Wu et al., 2014; Niu et al., 2017), indicative of the successful synthesis of Zn/Co-MOF. When the Zn/Co-MOF was carbonized at 900°C and rinsed with acid and water, nanoporous carbon was obtained, for the XRD pattern of MOF-NPC exhibits the presence of the two typical broad peaks rooting from the (002) and (101) planes at 2θ of 26° and 43°. The annealed products of Zn/Co-MOF at 450°C under N_2_ or N_2_-Air were also examined by XRD to verify their crystallographic structure and phases. From the XRD results, it can be concluded that ZnCo_2_O_4_ with high purity was obtained by annealing under N_2_ and in air. In the XRD patterns of ZnCo_2_O_4_, the strong and dominant diffraction peaks are located at 18.9°, 31.7°, 36.9°, 44.9°, 56.6°, 59.3°, and 65.2° and can be ascribed to the (111), (220), (311), (400), (422), (511), and (440) planes of the spinel ZnCo_2_O_4_ (JCPDS card No.23-1390). There are some weak peaks at 31.1°, 34.5°, 36.2°, 47.6°, and 62.9°, which can be indexed to the (100), (002), (101), (102), and (103) planes of ZnO. No other impurity peaks were observed. Compared with ZnCo_2_O_4_, the sample obtained without heat-treatment in air had faint diffraction peaks of ZnCo_2_O_4_, which indicated that the crystallographic structure of the oxides can't be formed well without heat-treatment in air due to the presence of carbon elements, so it was designated as C@ZnCo_2_O_4_ in this study.

**Figure 4:**
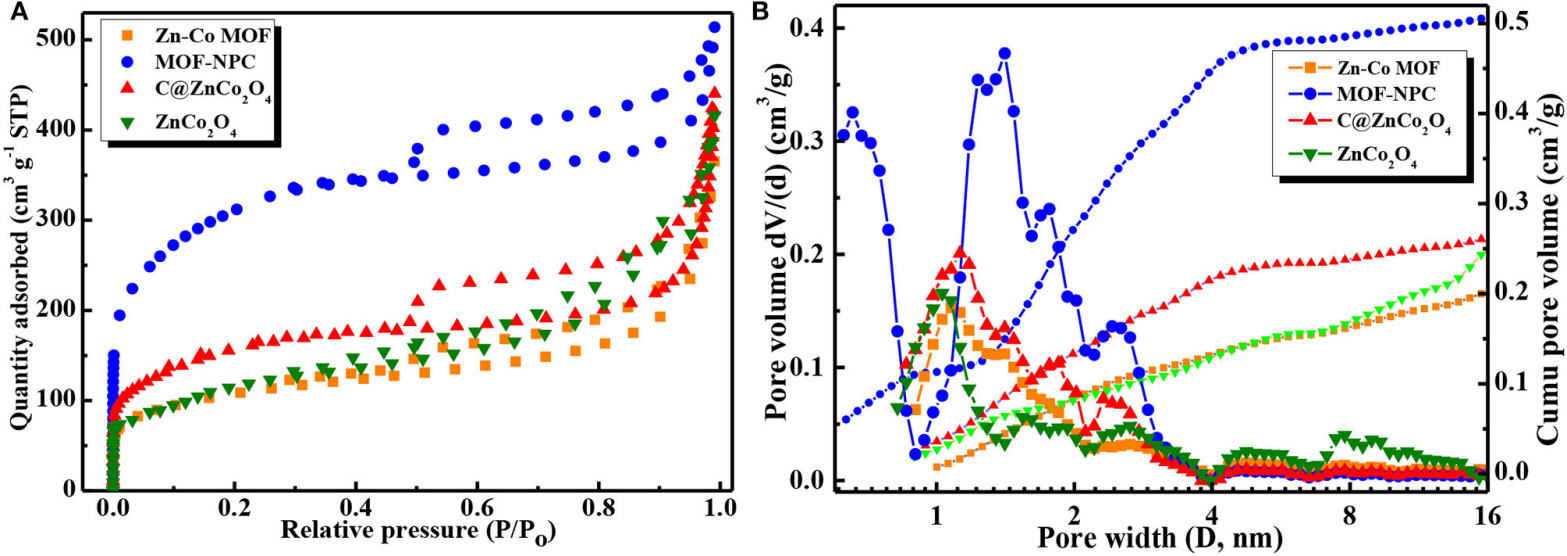
**(A)** N_2_ adsorption/desorption isotherms at 77K, **(B)** Pore size distribution (PSDs) of the Zn/Co-MOF and Zn/Co-MOF derived nanoporous carbon (NPC) and metal oxides calculated with NLDFT method.

The specific surface area and porosity of Zn/Co-MOF and Zn/Co-MOF derived nanoporous carbon (NPC) and metal oxides were characterized by nitrogen adsorption/desorption measurements at 77 K and pore size distributions (PSDs) analysis. As depicted in Figure 4A, all the samples show obvious hysteresis loop at the relative pressure range of 0.48–0.99 between the adsorption and desorption branches, so these isotherms can be classified into a type IV curve according to the IUPAC definition and classification (He et al., 2019, 2020). The presence of a hysteresis loop suggested that there are a number of mesopores in these samples. Comparatively speaking, the MOF-NPC has a significantly higher nitrogen adsorption amount than the other samples, especially in the low relative pressure range (P/P_o_) <0.02, which indicated that MOF-NPC possessed the largest SSA and pore volume with hierarchical porous structures. The PSDs of these samples were derived from the isotherms and calculated with NLDFT model (Li et al., 2018), and the results are presented in Figure 4B. The PSDs manifest that all the samples have hierarchical pore structures containing a large number of micropores, mesopores, and even macropores. This kind of hierarchically porous structure is conducive to energy storage, for the micropore is suitable for the ion accumulation and the mesopore is beneficial to the ion transportation (Ma et al., 2014).

The SSA and porosity parameters of Zn/Co-MOF, MOF-NPC, C@ZnCo_2_O_4_, and ZnCo_2_O_4_ were calculated using the Brunauer-Emmett-Teller (BET) and NLDFT, which is listed in Table 1. The SSA and pore volume of MOF-NPC is as high as 1,137 m^2^/g and 0.60 cm^3^/g, while the SSAs of the others are 577, 440, and 384 m^2^/g for C@ZnCo_2_O_4_, ZnCo_2_O_4_, and Zn/Co-MOF, respectively. These indicate that pores are formed in the metal organic frameworks, and nanoporous carbon and nanoporous metal oxides can be obtained from the single MOF using the “One-for-All” strategy. It is recognized that large SSA and high porosity are always correlated with the excellent electrochemical performance of electrode materials, so it is expected that the MOF-derived NPC and bimetallic oxides will display excellent electrochemical performances in supercapacitors.

**Table 1:** Textural properties of Zn/Co-MOF and its derivates calculated from N_2_ adsorption at 77 K.

**Samples**	**S_**BET**_ (m^**2**^ g^**−1**^)**	**V_**tot**_ (cm^**3**^ g^**−1**^)**	**V_**micro**_ (cm^**3**^ g^**−1**^)**	**V_**meso**_ (cm^**3**^ g^**−1**^)**	**V_**meso**_/V_**tot**_ (%)**
MOF-NPC	1,137	0.60	0.35	0.25	41.6
C@ZnCo_2_O_4_	577	0.40	0.17	0.23	57.5
ZnCo_2_O_4_	440	0.45	0.10	0.35	77.7
Zn/Co-MOF	384	0.38	0.11	0.27	71.0

**Figure 5:**
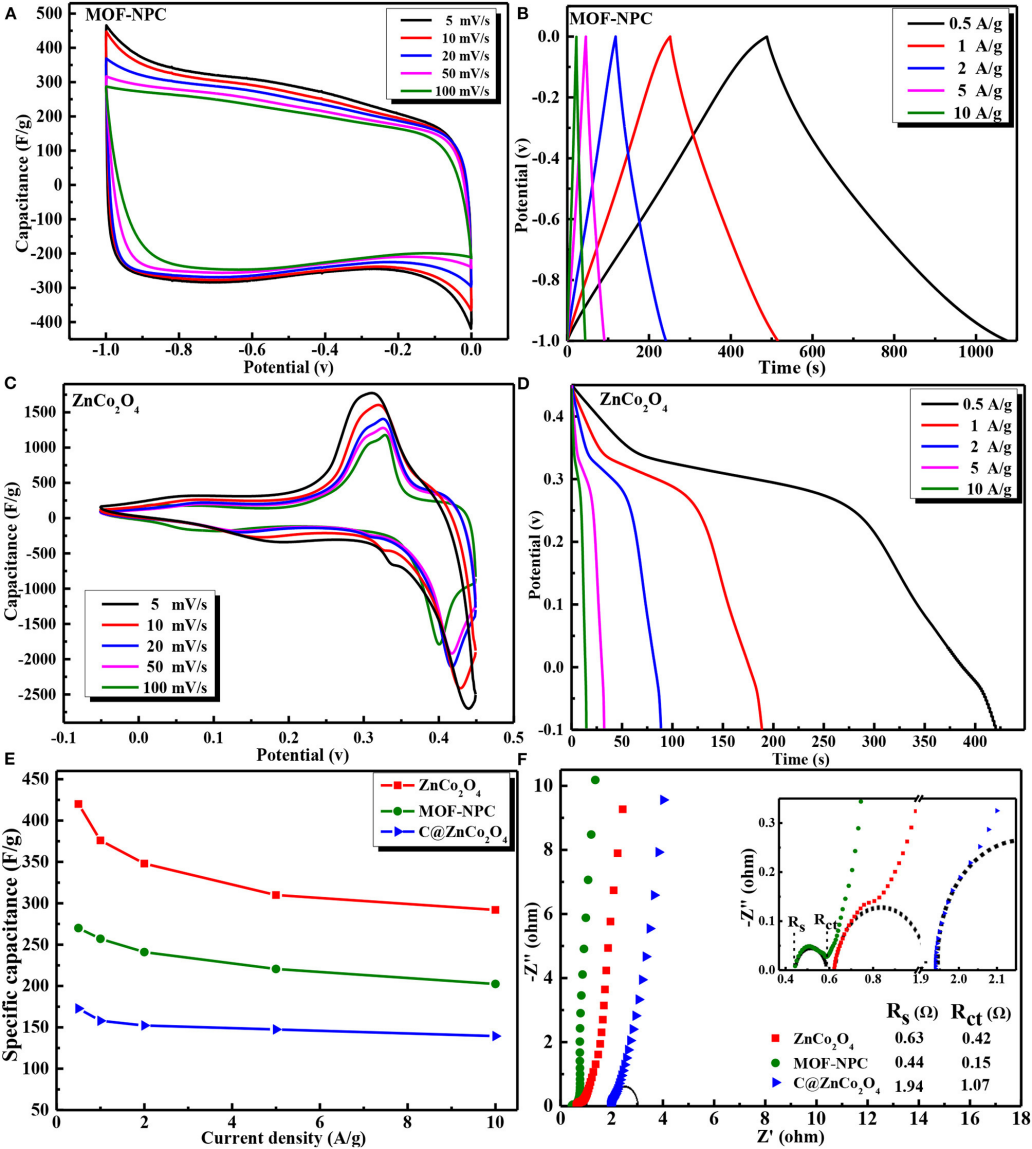
The three-electrode electrochemical performances of Zn/Co-MOF-derived NPC and metal oxides: CV curves at various scan rates of **(A)** MOF-NPC and **(C)** ZnCo_2_O_4_; GCD curves at various current density of **(B)** NPC and **(D)** ZnCo_2_O_4_; **(E)** Specific capacitance at different current density of MOF-NPC, C@ZnCo_2_O_4_, and ZnCo_2_O_4_; **(F)** EIS curves of MOF-NPC, C@ZnCo_2_O_4_, and ZnCo_2_O_4_.

The as-obtained Zn/Co-MOF-derived nanoporous carbon and bimetallic oxides were examined as electrode materials for supercapacitor applications. To systematically investigate the electrochemical performances of the NPC and ZnCo_2_O_4_ electrodes, cyclic voltammetry (CV) and galvanostatic charge–discharge (GCD) tests were firstly carried out in a three-electrode system with an aqueous 6 M KOH as electrolyte. The CV studies for the NPC-based and the nanoporous ZnCo_2_O_4_-based electrodes were tested in the potential window of −1.0 to 0.0 V and −0.05 to 0.45 V (vs. Hg/HgO), respectively. Figures 5A,C present the capacitive behaviors of the Zn/Co-MOF derived NPC and ZnCo_2_O_4_ at different scan rates from 5 to 100 mV/s. The CV curves of the NPC are very close together and their rectangular symmetry can still be maintained at a high scan rate of 100 mV/s without obvious change and distortion, indicating that the NPC has an excellent rate capability. The specific capacitance of the NPC-based electrode calculated by integrating the CV curves can reach 270 F/g at the scan rate of 5 mV/s, and still keep 202.4 F/g even at 100 mV/s. Its high rate performance is ascribed to its large amount of mesopores generated from the unique MOF structure, which facilitates the electrolyte ions transfer in the pore channels. In the case of nanoporous ZnCo_2_O_4_-based electrodes, it is clearly seen in Figure 5C that all CV curves show a pair of obvious redox peaks, indicating the typical faraday properties of battery-type electrodes. Calculating from the CV curves, the specific capacitances of the ZnCo_2_O_4_-based electrodes are 508 and 309.2 F/g as the scan rate is 5 and 100 mV/s.

The GCD curves of the NPC- and ZnCo_2_O_4_-based electrodes at various current densities from 0.5 to 10 A/g are displayed in Figures 5B,D. As for NPC, the GCD curves have no obvious IR drop at the initial discharge stage and always retain symmetric triangular shapes at all the current densities, indicating the electrical double-layer capacitor of carbon materials. The GCD and CV results confirmed that the MOF-derived NPC has outstanding columbic efficiency and desirable capacitive behavior when it was used as supercapacitor electrode. For the nanoporous ZnCo_2_O_4_, it is clear that potential plateaus exist in the GCD curves at all the current densities, suggesting its battery-like feature, which is consistent with the CV results. The probable electrochemical redox is associated with the Co-O/Co-O-OH conversion in alkaline electrolyte (Venkatachalam et al., 2017; Xu et al., 2017a), and the reactions can proceed as **Equations (5), (6)**:

(5) $$\begin{array}{l} ZnCo_{2}O_{4}+H_{2}O+OH^{-}⇆ZnOOH+2CoOOH+e^{-} \end{array}$$

(6) $$\begin{array}{l} Co{\left(OH\right)}_{2}+H_{2}O+e^{-}⇆CoO_{2}+OH^{-} \end{array}$$

The mass-specific capacitance calculated from GCD curves according to **Equation (1)** at various current densities are plotted in Figure 5E. The specific capacitances of the NPC, ZnCo_2_O_4_, obtained with or without heat-treatment in air are presented in this figure. At the current density of 0.5 A/g, the specific capacitance of ZnCo_2_O_4_, NPC, and C@ZnCo_2_O_4_ is 420, 270, and 173 F/g. Obviously, the capacitance of NPC is higher than most carbons (Bi et al., 2019; Yin et al., 2020). In addition, the capacitance performance of ZnCo_2_O_4_ is much better than that of C@ZnCo_2_O_4_, because the latter contains a large amount of organic carbons that reduces its conductivity, resulting in poor electrochemical performance.

In order to further understand the electrochemical performance of ZnCo_2_O_4_, C@ZnCo_2_O_4_, and NPC-based electrodes, the electrical impedance spectroscopy (EIS) measurements were conducted and presented in Figure 5F. All these Nyquist plots are composed of high frequency semicircles and low frequency Warburg steep lines, which correspond to the charge transfer limiting process and diffusion limiting electrode process. In the high frequency region, the intercept at the real part Z' gives the internal resistance (***R***_***s***_), which is the sum of the intrinsic resistance of electrode materials, the contact resistance at the interface of active material/current collector, and the ionic resistance of the electrolyte (Zheng et al., 2016). The diameter of the semicircle represents the charge-transfer resistance (***R***_***ct***_) resulted from the Faradaic reactions. The fitted *R*_*s*_ and ***R***_***ct***_ values of the three electrodes are inset in Figure 5F. It is clear that the NPC-based electrode has the lowest ***R***_***s***_ and ***R***_***ct***_ of 0.44 Ω and 0.15 Ω, and C@ZnCo_2_O_4_-based electrode has the maximal resistances at almost three times that of the ZnCo_2_O_4_ electrode. This further confirmed that it is necessary to anneal the MOF in air for some time to obtain metal oxides with good electrochemical performance.

In order to further estimate the feasibility of the practical applications of the Zn/Co-MOF-derived materials in electrochemical energy storage, the capacitive performance of asymmetric supercapacitors (ASCs) were investigated in which the NPC acted as the negative and ZnCo_2_O_4_ as the positive electrode materials. Simultaneously, the symmetric supercapacitors (SSCs) cell was assembled based on the single NPC or ZnCo_2_O_4_, which were fabricated and evaluated for comparison. These SCs were constructed using cellulose papers as separators in 6 M KOH aqueous electrolyte. The capacitive performances of these devices were also tested by CV curves, GCD, and EIS measurements. According to the preliminary experiments, the ASC device has far superior capacitive performance to the other two SSCs, so only the CV and GCD results of the ASCs device are presented here.

**Figure 6:**
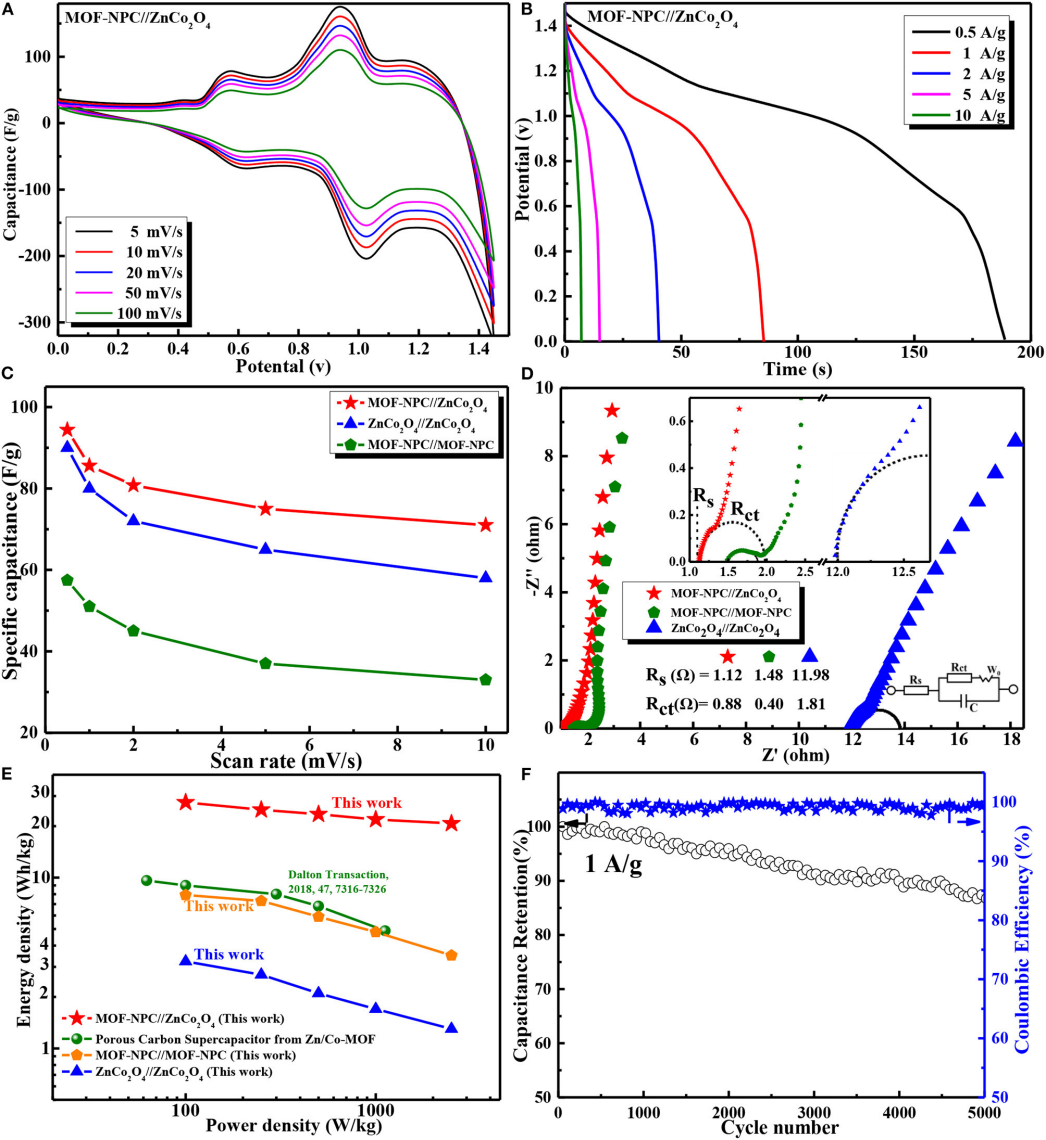
Electrochemical performances of asymmetric and symmetric supercapacitors in a two-electrode configuration with aqueous 6 M KOH solution as electrolyte: **(A)** CV curves at various scan rates; **(B)** GCD curves at different current densities of asymmetric supercapacitor; **(C)** Specific capacitance of cells at different current densities; **(D)** EIS curves of various supercapacitor; **(E)** Ragone plots of the assembled supercapacitors in this study and the reported in literatures; **(F)** Cycling performance and coulombic efficiency of the device at 1 A/g. The inset is a photograph of LED lamp powdered by the assembled ASC CR2025 coin cell.

The CV curves of ASCs (MOF-NPC//ZnCo_2_O_4_) were obtained in a voltage window ranging from 0.0 to 1.45 V at various scan rates (shown in Figure 6A). In contrast, the voltage window of the symmetric SCs assembled with MOF-NPC//MOF-NPC and ZnCo_2_O_4_//ZnCo_2_O_4_ is 0.0 to 1.0 V and 0.0 to 0.45 V. Obviously, the voltage window of the ASCs matches well with the working voltage windows of the corresponding individual electrodes; this is due to the unique advantages of the “*One-for-All*” strategy in preparing electrode materials derived from the same precursor (He et al., 2019; Jia et al., 2019). All the CV curves of the ASCs display a couple of reversible redox peaks between 1.1 and 0.8 V at different scan rates within the working voltage of 1.45 V, which demonstrated that the intensive ASC-type capacitance primarily was contributed to from both the negative and positive electrodes. GCD measurements at a potential window from 0.0 to 1.45 V were also conducted at various current densities from 0.5 to 10 A/g and shown in Figure 6B. The initial voltage loss (IR drop) is negligible in the discharge curves, even at the high current density of 10 A/g, suggesting a fast ***I-V*** response and very low internal resistance of the ASCs (Salunkhe et al., 2015a), which will be confirmed by the EIS results. Moreover, a sloping discharge pseudo-plateau was observed in the voltage range from 1.1 to 0.8 V, which is consistent with the CV results, and this further verified that the ASCs had a battery-like supercapacitor behavior.

The specific capacitance of these SCs devices were calculated by **Equation (2)**, including the SSCs (MOF-NPC//MOF-NPC, ZnCo_2_O_4_//ZnCo_2_O_4_) and ASCs(MOF-NPC//ZnCo_2_O_4_). Figure 6C shows the specific capacitances of these devices at different scan rates. It is apparent that the ASCs have much higher specific capacitance than that of the SSCs; the specific capacitance of ASCs can arrive up to 94.4 F/g at a scan rate of 5 mV/s and 71.0 F/g at 100 mV/s. The EIS of the devices were also tested and shown in Figure 6D with an inset of the equivalent circuit diagram. ***R***_***s***_ values of 1.12, 1.48, and 11.98 Ω can be obtained for ASCs, MOF-NPC//MOF-NPC, and ZnCo_2_O_4_//ZnCo_2_O_4_ SCs, respectively. The ASCs have the lowest internal resistance due to the exact compatibility, and the medium charge-transfer resistance (***R***_***ct***_) of 0.88 Ω, which reveals its outstanding electrical conductivity, highly effective ion diffusion, and rapid charge transfer in the charge/discharge process.

Moreover, Ragone plot (power ***vs*** energy density) is adopted to evaluate the performance of the as-assembled ASC, since it is a direct performance indicator for energy storage devices. The Ragone plots of the ASC and SSCs are depicted in Figure 6E, including those of similar devices reported in the literature. The energy density (E_cell_) and power density (P_cell_) are calculated using the **Equations (3), (4)**. It is found that the ASCs (MOF-NPC//ZnCo_2_O_4_) can deliver a maximum energy density of 28.6 Wh/kg at a power density of 100 W/kg, and still remain 18 Wh/kg at 2.5 kW/kg. By contrast, the two SSCs have a much lower energy density at the same density; the maximal energy density of the ZnCo_2_O_4_ SSC is just 8 Wh/kg, although it has comparable specific capacitance with that of the ASC due to its narrow voltage window of 0–0.45 V. The Ragone plots of NPCs-based SSCs almost overlap with that of SSCs assembled with porous carbon nanofibers derived from similar Zn/Co-MOF (Yao et al., 2018) in the literature, whose energy density is only 9.61 Wh/kg at a power density of 62 W/kg and 4.88 Wh/kg at 1.1 kW/kg even in organic electrolytes with a high voltage window of 2.5 V. These demonstrated that the ASCs have superior performance to SSCs.

The superior energy storage performance of the ASCs in this study are mainly attributed to the following practical reasons: (1) Both the NPC and ZnCo_2_O_4_ electrode materials derived from the same MOF precursor show a hierarchically porous structure with a large specific surface area and an appropriate ratio of mesopore to micropore, which provide not only abundant active sites for ion accumulation but also pore channels for the rapid ion transfer; (2) The electrode materials have a nanometer size, so the charge transport path during the charging and discharging process is short, leading to excellent rate performance; and (3) The ASCs have a wide working voltage window of 1.45 V that is equal to the sum of the individual NPC and ZnCo_2_O_4_ electrode, while the matchable voltage window and specific capacitance remarkably enlarge the energy density of the ASCs. The cycling performance of the MOF-NPC//ZnCo_2_O_4_ ASCs was inspected at a current density of 1 A/g shown in Figure 6F. It can also be seen that after 5,000 charge-discharge cycles, the specific capacity retention rate of the ASCs is about 87.2% of its initial value, implying the excellent cycle performance of the cell. These results confirmed that the ASCs assembled with the NPC and metal oxides derived from the same MOF precursor had promising potentials in the field of practical energy storage.

## Conclusions

In summary, a facile “One-for-All” strategy is adopted to obtain both negative/positive electrode materials for ASCs from a single bimetal-organic framework, in which the NPC and ZnCo_2_O_4_ are derived from Zn/Co-MOF as the single precursor. The conversion process for preparing NPC and ZnCo_2_O_4_ was optimized. ZnCo_2_O_4_ with higher phase purity can be obtained by annealing Zn/Co-MOF at 450°C first under N_2_ followed by annealing in air for some time. Owing to the unique framework structure, the derived negative (NPC) and positive (bimetal oxides) electrode materials display a large specific surface area and high pore volume with hierarchically porous structures. Symmetric and asymmetric supercapacitors were assembled using the as-prepared NPC and ZnCo_2_O_4_, and their electrochemical performance were evaluated by CV, GCD, and EIS measurements. Benefitting from the hierarchical porous structures and the matchable voltage window and specific capacitance of the negative and positive electrode, the ASCs exhibit an excellent battery-like capacitive performance and rate capacity, which is far superior to that of SSCs based on NPC or ZnCo_2_O_4_. The ASCs can deliver a maximum energy density of 28.6 Wh/kg at a power density of 100 W/kg, and still remain 18 Wh/kg at 2.5 kW/kg, and it shows excellent cycling stability.

## Data Availability Statement

The raw data supporting the conclusions of this article will be made available by the authors, without undue reservation.

## Author Contributions

DH: formal analysis, methodology, and writing—original draft. YG: methodology. YY: resources and methodology. LW: resources and supervision. JZ: supervision. Z-HH: project administration. M-XW: conceptualization, resources, writing—review and editing, supervision, and funding acquisition. All authors contributed to the article and approved the submitted version.

## Conflict of Interest

The authors declare that the research was conducted in the absence of any commercial or financial relationships that could be construed as a potential conflict of interest.
